# Prediction of Remnant Liver Regeneration after Right Hepatectomy in Patients with Hepatocellular Carcinoma Using Preoperative CT Texture Analysis and Clinical Features

**DOI:** 10.1155/2021/5572470

**Published:** 2021-06-10

**Authors:** Tong Zhang, Yi Wei, Xiaopeng He, Yuan Yuan, Fang Yuan, Zheng Ye, Xin Li, Hehan Tang, Bin Song

**Affiliations:** ^1^Department of Radiology, Sichuan University West China Hospital, Chengdu 610041, Sichuan Province, China; ^2^Department of Radiology, Affiliated Hospital of Southwest Medical University, Luzhou 646000, Sichuan Province, China; ^3^GE Healthcare Research, Nanjing 210000, China

## Abstract

**Objectives:**

To predict the regenerative rate of liver in patients with HCCs after right hepatectomy using texture analysis on preoperative CT combined with clinical features.

**Materials and Methods:**

88 patients with 90 HCCs who underwent right hepatectomy were retrospectively included. The future remnant liver was semiautomatically segmented, and the volume of future remnant liver on preoperative CT (LV_pre_) and the volume of remnant liver on following-up CT (LV_fu_) were measured. We calculated the regeneration index (RI) by the following equation: (LV_fu_ – LV_pre_)/LV_pre_) × 100 (%). The support vector machine recursive method was used for the feature selection. The Naive Bayes classifier was used to predict liver RI, and 5-fold cross-validation was performed to adjust the parameters. Sensitivity, specificity, and accuracy were calculated to evaluate the diagnostic efficiency of the model.

**Results:**

The mean RI was 142.99 ± 92.17%. Of all clinical parameters and texture features, the AST, ALB, PT-INR, Perc.10%, and S(5, −5)Correlat were found to be statistically significant with RI. The diagnostic sensitivity, specificity, and accuracy of the model in the training group were 0.902, 0.634, and 0.768, and the AUC value of the obtained model was 0.841. In the test group, the sensitivity, specificity, and accuracy of the model were 1.0, 0.429, and 0.778, respectively, and the AUC value was 0.844.

**Conclusion:**

The use of texture analysis on preoperative CT combined with clinical features can be helpful in predicting the liver regeneration rate in patients with HCCs after right hepatectomy.

## 1. Introduction

Hepatocellular carcinoma (HCC) is the fifth most common type of cancer and the second leading cause of cancer-related death worldwide [1]. The treatments of HCC are varied, among which surgical excision is the main method of HCC therapies [2]. Although the remnant liver tissue after partial hepatectomy has great regenerative potential and even back to its original size [3], posthepatectomy liver failure in patients is still the leading cause of liver excision-related mortality [4, 5], with an incidence of between 1.2% and 32% [6]. This may be explained by the fact that the remnant liver volume is inadequate after hepatectomy and cannot meet the body's normal metabolic needs and may directly lead to liver dysfunction, liver failure, and even death [7–9]. In addition, the remnant liver volume can not only directly reflect the amount of the normal hepatic cells, but provide the chance to further evaluate the hepatic function [10, 11]. Thus, accurate estimation of remnant liver volume and evaluation of remnant liver regenerative ability after surgery are of pivotal importance in avoiding postoperative liver insufficiency and even liver failure.

At present, multiple imaging modalities have been explored to measure the volume of remnant liver, including computed tomography (CT), magnetic resonance imaging (MRI), and ultrasound [4, 12]. Of these imaging modalities, the volumetric CT plays an important role in evaluating the liver volume as it has good reconstructive ability [13, 14]. Furthermore, the texture analysis based on CT images also presented a promising future because of its ability to assess tissue heterogeneity. Regarding the texture analysis, it described a wide range of techniques that enable quantification of the gray-level patterns, pixel interrelationships, and spectral properties of an image [15] and demonstrated a higher sensitivity to the heterogeneity perception of the lesion relative to human visibility analysis [15–18]. Kim et al. [13] investigated the use of CT texture analysis in liver regeneration prediction and found that texture analysis can be useful in predicting the liver regeneration rate in patients with liver transplantation. To our knowledge, few studies have investigated the relationship between the results of CT texture analysis and rate of liver regeneration after right hepatectomy in patients with HCCs.

Therefore, the purpose of this study was to predict the rate of liver regeneration in patients with HCCs after right hepatectomy using texture analysis on preoperative CT combined with clinical features.

## 2. Materials and Methods

### 2.1. Patients

**Figure 1:**
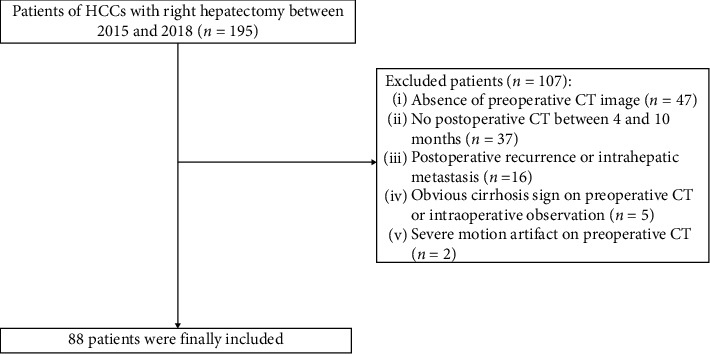
Flowchart of the participants with HCCs in this study. From 2015 to 2018, 195 patients were potentially enrolled. In addition, 107 patients were excluded because of the exclusion criteria; thus, 88 patients were finally included.

This retrospective study was approved by our institutional review board, and the requirement for patient consent was waived. This study was conducted in accordance with the 1964 Helsinki Declaration. From December 2015 to May 2018, a total of 195 consecutive patients who underwent right hepatectomy were enrolled in the initial population. All included patients were confirmed by surgical pathology. Among these patients, 107 were excluded because of the exclusion criteria (Figure 1). Consequently, 88 patients with 90 HCCs who underwent right hepatectomy were included for analysis (mean age 50.25 ± 11.57 years; range 23–78 years) with 90 lesions, including 79 men (50.76 ± 12.03 years; range 23–78 years) and 9 women (45.78 ± 4.24 years; range 39–52 years). The baseline characteristics including sex, age, alanine aminotransferase (ALT), aspartate aminotransferase (AST), alkaline phosphatase (ALP), gamma-glutamyl transpeptidase (GGT), total protein (TP), albumin (ALB), total bilirubin (TBIL), direct bilirubin (DBIL), hemoglobin, platelet (PLT), and prothrombin time-international normalized ratio (PT-INR) were collected.

### 2.2. CT Techniques

Patients underwent contrast-enhanced CT examinations using one of the following systems: Revolution CT (GE Healthcare) or SOMATOM definition (Siemens). The CT examination included three phases: precontrast, arterial, and portal vein phase. Arterial phase scanning started about 30–35 s after the beginning of injection, and portal phase was obtained after 2 min with the contrast injection. The following parameters were used: tube voltage, 120 kVp or 100 kVp; tube current, 200–450 mA; slice thickness, 1.25 mm; pitch, 0.992 : 1; rotation speed: 0.5 s/rot; ASIR-V: 20%. All patients received an intravenous, nonionic contrast medium (iodine concentration, 370 mg/mL; volume, 1.5–2.0 ml/kg of body weight; contrast type, Iopromide Injection, Bayer Pharma AG) at a rate of 2-3 ml/s, and after contrast agent injection, a 20 ml saline was injected for the flush.

### 2.3. Preoperative CT Texture Analysis

We applied MaZda (version 4.6) [19, 20] for liver segmentation with texture features extraction. The portal phase images of the preoperative CT examination of the patients with HCCs were used for the texture analysis.

#### 2.3.1. Image Selection

The images of a relative maximum axial section of the running trajectory of the middle hepatic vein on portal phase were chosen for texture analysis.

#### 2.3.2. ROI Definition

A freehand ROI was drawn to outline the margin of the liver, which was about 1-2 mm from the liver border (to minimize the volume effect). Additionally, the virtual surgical resection of the liver was along the inner side of the middle hepatic vein, which demarcates the right lobe from the future remnant liver. The border of the liver is defined as the perihepatic fat tissue between the liver and the adjacent tissues. At the same time, the inferior vena cava, hepatic vein, portal vein, and their main branches, gallbladder, or calcification were excluded from ROIs to include only hepatic parenchyma as much as possible.

#### 2.3.3. Data Standardization

To minimize the effects of variations of contrast and brightness of images, the gray-level values of pixels were normalized by putting into the range of *μ* ± 3*σ* (*μ*, mean gray-level value; *σ*, standard deviation) before statistical analysis.

#### 2.3.4. Texture Feature Extraction

The texture features were extracted by MaZda and primarily came from gray-level histogram (GLH), gray-level co-occurrence matrix (GLCM), gray-level run long matrix (GLRLM), histogram of oriented gradient (HOG), wavelet transformation (WT), and autoregressive model (ARM). Finally, 281 texture features [20] were extracted from the ROIs of the future remnant liver parenchyma.

### 2.4. Preoperative CT Liver Volume

The volume of future remnant liver (LV_pre_) on preoperative CT was calculated by the software (UNITED IMAGING Workstation). The portal phase images of the preoperative CT scan were selected for volume analysis.

After the CT images were loaded into the software, the entire liver and vessels in the liver (mainly including hepatic portal vein, hepatic vein, and their main branches) were automatically segmented. Among a set of CT section images of each patient, the images of the maximum axial section of each HCC on portal phase were chosen and the tumors were semiautomatically segmented by a radiologist (with 7 years of abdominal CT clinical experience). After segmenting the liver and tumors, the radiologist drew a virtual surgical plain along the middle hepatic vein with reference to the operative record and the postoperative CT scan. Then, the software automatically calculated the volume of the function future graft (LV_pre_) (LV_pre_ = future graft volume − vessel volume inside future graft).

### 2.5. Postoperative CT Liver Volume

**Figure 2:**
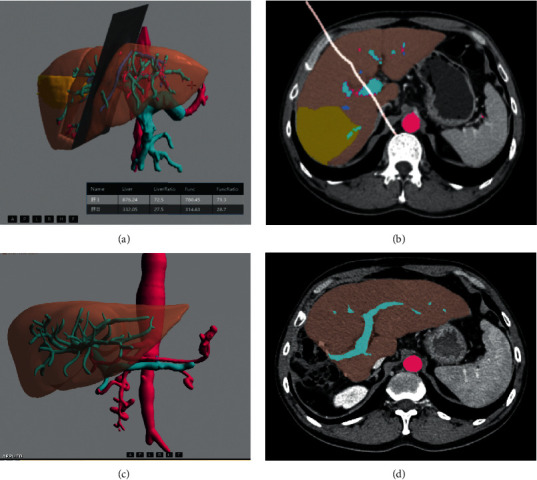
A 51-year-old male patient with HCC underwent CT scan of the portal venous phase before and after regular right hepatectomy. (a) 3D image of the preoperative simulated surgical tangent (approximately traveling along the middle hepatic vein). The functional liver volume of the future liver is automatically displayed in the lower right of the figure. (b) The axial image of the cross section of the preoperative simulated surgical tangent. (c) 3D images of actual remnant liver on the sixth month after surgery. (d) The axial image of the actual remnant liver on the sixth month after surgery.

As our study was retrospective, the timing of postoperative follow-up CT was varied. Previous study has shown that, after right trisegmentectomy of the liver, the remnant liver tissue began to regenerate 5–10 days after the operation, and the original size and function could be completely restored in about 6 months [21]. The postoperative CT images on portal phase performed between 3 and 10 months were used to calculate the volume of remnant liver (LV_fu_). In the case of more than one CT examination during the time above, the images closest to the 6^th^ month were chosen for LV_fu_ calculation. The procedure of remnant liver and vessel segmentation on postoperative CT images was the same as described on preoperative CT images (Figure 2). Then the LV_fu_ was automatically calculated. Therefore, we calculated the regeneration index (RI) with these obtained volumetric parameters depending on the following equation: ((LV_fu_–LV_pre_)/LV_pre_) × 100 (%). In addition, the cutoff value of RI was determined as 100%, which means that the value higher than 100% was higher RI group and less than 100% was lower RI group [13].

### 2.6. Statistical Analysis

*χ* ^2^ or Fisher's exact test was used for the categorical variables. Continuous variables were firstly checked for homogeneity by using *F* test, and independent sample test or *t*′ test was used for the continuous variables. Stratified sampling method was used for training and test set determination. There were 293 features (including 281 texture features and 12 clinical factors) for building prediction model. We first calculated the Spearman correlation coefficient to remove high-correlation features with a threshold of 0.8+ and then used the support vector machine recursive method for feature selection. The Naive Bayes classifier was used to predict liver RI, and 5-fold cross-validation was performed to adjust the parameters. Sensitivity, specificity, and accuracy were used to evaluate the diagnostic efficiency of the model. At the same time, the test set was brought into the Naive Bayes classifier to establish the diagnostic efficiency of the test set. *p* values less than 0.05 were considered statistically significant. Statistical analysis was performed using SPSS 17.0 (SPSS Inc., Chicago) and R software.

## 3. Results

### 3.1. Demographic Characteristics and Laboratory Findings

The demographic characteristics and preoperative laboratory findings of the patients are summarized in Table 1. Of all these clinical parameters, only the AST (*p*=0.018), ALB (*p*=0.024), and PT-INR (*p*=0.008) were found significantly different between the higher RI and lower RI group. Additionally, no statistically significant difference was found between the higher RI and lower RI group with sex, age, ALT, ALP, GGT, TP, TBIL, DBIL, hemoglobin, and PLT (all *p* > 0.05). The LV_pre_ was 367.14 ± 95.61 cm^3^ (the higher RI group) versus 541.45 ± 117.52 cm^3^ (the lower RI group); the LV_fu_ was 1009.72 ± 176.63 cm^3^ (the higher RI group) versus 928.21 ± 204.81 cm^3^ (the lower RI group); and the RI was 191.64 ± 90.80% (the higher RI group) versus 72.72 ± 22.51% (the lower RI group).

### 3.2. Texture Features for Predicting Liver Regeneration

**Figure 3:**
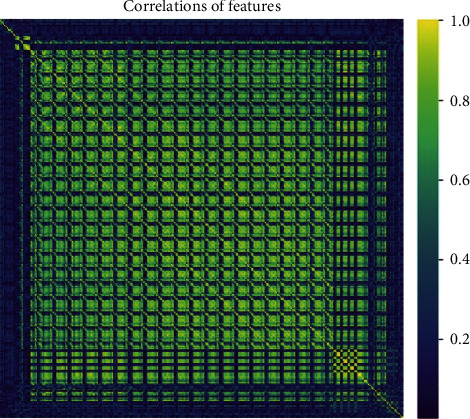
This is correlation map of features in which *X* and *Y* axes represent every feature's correlation coefficient ranging from 0 to 1 (see the bar indicator on right side). The heat map normally indicates the dependence distribution of extracted feature.

A total of 32 texture features were selected for model building. Heat map of correlations of features was shown in Figure 3. Of all these texture features, only the Perc.10% (*p*=0.039) and S(5, −5)Correlat (*p*=0.016) were found significantly different between the higher RI and lower RI group. Additionally, no statistically significant difference was found between the higher RI and lower RI group with the other texture features (all *p* > 0.05). The results of the texture analysis are summarized in Table 2.

### 3.3. Prediction Model for Liver Regeneration

**Figure 4:**
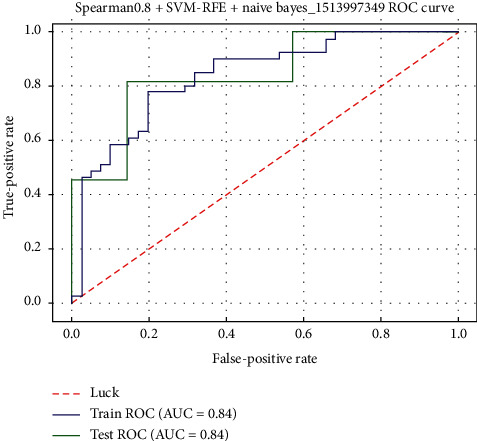
The ROC diagnosis curve of the training and test set.

The diagnostic sensitivity, specificity, and accuracy of the model in the training group were 0.902, 0.634, and 0.768 to differentiate the higher and lower regeneration group, and the AUC value of the obtained model was 0.841. In the test set, the sensitivity, specificity, and accuracy of the model were 1.0, 0.429, and 0.778, respectively, and the AUC value was 0.844. The ROC diagnosis curve of the training and test set was shown in Figure 4.

## 4. Discussion

In this study, we predicted the rate of liver regeneration in patients with HCCs after right hepatectomy using texture analysis on preoperative CT combined with clinical features. Our results showed that the mean RI was 142.99 ± 92.17%, with highly variable range from 16.0% to 597%. Of all clinical parameters and texture features, the AST, ALB, PT-INR, Perc.10%, and S(5, −5)Correlat were found statistically significant between the higher RI and lower RI group. Additionally, the model showed high diagnostic performance in differentiating the higher and lower RI group.

Comparing the clinical parameters between the higher and lower RI group, the AST, ALB, and PT-INR were relevant factors affecting remnant liver regeneration. AST is mainly present in the mitochondria of hepatocytes, and advanced liver disease may be associated with mitochondrial injury. In addition, progression of liver fibrosis may reduce the clearance of AST, leading to increase of serum AST level [22, 23]. Previous studies have shown that AST is a common indicator to establish predictive models of liver disease [22, 24] and helps to assess the severity of liver cirrhosis [25]. Therefore, AST is elevated, and liver function is impaired, which may lead to a decreased ability of liver regeneration. For patients with hepatic fibrosis or cirrhosis, the reduction of effective hepatocytes will lead to a decrease in the level of ALB and II, V, VII, and X coagulation factors synthesized only in hepatocytes. PT-INR is used to better reflect the levels of coagulation factors above; thus ALB and PT-INR are important indicators to determine the severity and prognosis of the disease [26]. In this study, the ALB level and PT-INR in the higher regeneration group were significantly higher than those in the lower one probably because the impaired liver synthesis function would affect the liver regeneration process. Furthermore, the LV_pre_ was shown to be significantly lower in the higher RI group, which was consistent with previous study [13], which might be explained by the fact that the resection of larger volume of liver might result in more effective hepatic proliferation.

Comparing the texture features between the higher and lower RI group, S(5, −5)Correlat and Perc.10% were relevant factors affecting remnant liver regeneration. Texture refers to quantitative measures of spatial neighborhood interactions between pixel intensities within local neighborhoods in an image [27]. The most common method for assessing texture is the statistical method, and the run length matrix (RLM) and gray-level co-occurrence matrix (GLCM) are types of the statistical approach. The texture features from GLCM analyses represent the spatial distribution of gray levels, which indicates how often a pixel of one gray level is found with a certain relationship to another gray-level pixel [13]. The correlation refers to the degree of approximation of the matrix elements in space in the row or column direction. Therefore, the correlation is a reflection of the gray correlation of the local area in the image; that is, when the values of the matrix elements are uniformly equal, the correlation value is large; on the contrary, if the values of the matrix elements differ greatly, the correlation value is small. In this study, the value of S(5, −5)Correlat was higher in higher RI group than that in lower RI group. The parameter mainly reflects the heterogeneity of the liver parenchyma, and higher S(5, −5)Correlat value demonstrates a good correlation with the microscopic heterogeneity and may directly influence the liver regeneration, as good microscopic heterogeneity may represent a better liver regeneration. Additionally, the value of Perc.10% which derived from gray-level histogram was found higher in the higher RI group. Previous studies have shown the applications of histogram on various tumors [28–30]. However, the relationship between increased Perc.10% and liver regeneration has not been identified; further research needs to be done to identify the histopathological significance of our results.

In this study, in addition to the clinical characteristics, the texture parameters were also derived for the construction of the liver regenerative prediction model. The texture parameters entail the ability to better characterize the heterogeneity of the liver parenchyma. Thus, the model which contains both clinical and texture parameters can better describe the microenvironment of the normal liver tissue and further to better provide the predictive value for liver regeneration.

Our study had several limitations. First, a selection bias may have been present due to the single-center, retrospective design. Second, we only investigated the CT texture and laboratory test results with the relation to liver regeneration, but the pathological parameters such as the liver fibrosis, tumor differentiation grade, and even microvascular invasion were not considered as the retrospective design; thus, we should further prospectively collect more patients that contain the comprehensive information. Third, due to the variety of CT examination equipment included in this study, the CT scanning procedures are not completely consistent. Different CT equipment, tube current, scanning layer thickness, and reconstruction techniques all may affect the texture features. Therefore, in the following research, prospective study is needed to unify the scanning equipment for better research results.

In conclusion, the use of texture analysis on preoperative CT combined with clinical features can be helpful in predicting the liver regeneration rate in patients with HCCs after right hepatectomy.

## Figures and Tables

**Table 1:** Comparisons of clinical parameters between higher and lower RI groups.

Characteristics	Total (*N* = 88)	Higher RI (*N* = 52)	Lower RI (*N* = 36)	*t* value	*p* value
Sex					
Male	79	46	33	⋯	0.732
Female	9	6	3	⋯	
Age (years)	50.25 ± 11.57	50.50 ± 12.97	49.89 ± 9.34	0.242	0.809
ALT (IU/L)	59.88 ± 54.27	50.79 ± 50.30	73.00 ± 57.76	−1.916	0.059
AST (IU/L)	64.94 ± 54.67	53.52 ± 42.32	81.44 ± 65.92	−2.421	0.018^*∗*^
ALP (IU/L)	127.16 ± 72.90	129.00 ± 87.11	124.50 ± 46.24	0.283	0.778
GGT (IU/L)	138.46 ± 135.62	128.98 ± 147.67	152.14 ± 116.70	−0.786	0.434
TP (g/L)	71.69 ± 6.18	71.18 ± 5.78	72.43 ± 6.73	−0.939	0.351
ALB (g/L)	41.50 ± 4.30	42.35 ± 4.23	40.26 ± 4.15	2.299	0.024^*∗*^
TBIL (umol/L)	17.24 ± 8.35	17.59 ± 7.46	16.74 ± 9.58	0.470	0.640
DBIL (umol/L)	6.70 ± 3.77	6.80 ± 4.34	6.56 ± 2.80	0.286	0.776
Hemoglobin (g/L)	142.07 ± 17.57	143.37 ± 13.50	140.19 ± 22.25	0.831	0.408
PLT (10^9/L)	158.32 ± 65.03	157.64 ± 55.01	159.31 ± 78.11	−0.118	0.906
PT-INR	1.08 ± 0.11	1.05 ± 0.08	1.12 ± 0.14	−2.727	0.008^*∗∗*^
Liver volume (cm^3^)					
LV_pre_	438.45 ± 135.44	367.14 ± 95.61	541.45 ± 117.52	−7.650	＜0.001^*∗∗*^
LV_fu_	976.38 ± 191.80	1009.72 ± 176.63	928.21 ± 204.81	1.993	0.049^*∗*^
RI (%)	142.99 ± 92.17	191.64 ± 90.80	72.72 ± 22.51	7.684	＜0.001^*∗∗*^

*Note*. Data were presented as mean ± standard deviation. ALB: albumin; ALP: alkaline phosphatase; ALT: alanine aminotransferase; AST: aspartate aminotransferase; DBIL: direct bilirubin; GGT: gamma-glutamyl transpeptidase; PLT: platelet; PT-INR: prothrombin time-international normalized ratio; TBIL: total bilirubin; TP: total protein; LV_pre_: volume of future remnant liver; LV_fu_: volume of remnant liver; RI: regeneration index. ^*∗*^*p* < 0.05; ^*∗∗*^*p* < 0.01.

**Table 2:** Comparisons of texture features between higher and lower RI groups.

Texture features	Total (*N* = 88)	Higher RI (*N* = 52)	Lower RI (*N* = 36)	95% CI	*p* value
S(0, 3)DifVarnc	65.85 ± 11.89	64.52 ± 12.86	67.77 ± 10.20	[−8.36, 1.85]	0.209
WavEnHH_s-3	71.57 ± 16.89	68.91 ± 18.72	75.41 ± 13.14	[−13.69, 0.68]	0.076
WavEnLL_s-3	15137.44 ± 591.02	15170.74 ± 549.11	15089.33 ± 651.84	[−174.21, 337.03]	0.528
WavEnLH_s-5	435.40 ± 233.33.	401.95 ± 185.47	483.72 ± 284.85	[−181.39, 17.85]	0.106
WavEnHL_s-4	140.33 ± 54.38	136.97 ± 48.23	145.19 ± 62.60	[−31.72, 15.29]	0.489
Perc.10%	157.65 ± 19.97	154.00 ± 21.98	162.92 ± 15.45	[−17.36, −0.48]	0.039^*∗*^
WavEnLL_s-1	16977.39 ± 230.15	16982.18 ± 257.37	16970.46 ± 187.23	[−88.01, 111.46]	0.816
WavEnHL_s-3	117.75 ± 33.92	113.92 ± 36.33	123.30 ± 29.71	[−23.95, 5.18]	0.204
WavEnHH_s-4	70.75 ± 28.23	68.20 ± 27.35	74.44 ± 29.46	[−18.41, 5.93]	0.311
GrSkewness	0.97 ± 0.61	1.04 ± 0.71	0.87 ± 0.42	[−0.09, 0.44]	0.194
WavEnHL_s-5	186.59 ± 71.67	190.61 ± 76.56	180.77 ± 64.57	[−21.16, 40.84]	0.530
WavEnLL_s-5	10694.70 ± 1223.51	10766.76 ± 1137.15	10590.61 ± 1348.32	[−352.91, 705.22]	0.510
WavEnHH_s-5	96.30 ± 49.44	89.92 ± 46.84	105.50 ± 52.26	[−36.75, 5.59]	0.147
WavEnHL_s-2	174.42 ± 40.51	172.96 ± 45.21	176.52 ± 33.05	[−21.10, 13.99]	0.688
S(1, 0)SumVarnc	334.54 ± 44.08	333.92 ± 49.99	335.43 ± 34.47	[−20.61, 17.60]	0.876
S(4, 0)SumVarnc	226.84 ± 32.34	228.38 ± 35.43	224.62 ± 27.60	[−10.23, 17.76]	0.594
WavEnLH_s-4	308.90 ± 116.43	313.05 ± 125.10	302.91 ± 104.08	[−40.28, 60.57]	0.690
S(5, −5)Correlat	0.10 ± 0.08	0.12 ± 0.09	0.08 ± 0.05	[0.01, 0.07]	0.016^*∗*^
_MaxNorm	220.24 ± 26.44	217.65 ± 28.97	223.97 ± 22.16	[−17.70, 5.06]	0.273
S(0, 4)SumAverg	64.64 ± 0.57	64.68 ± 0.66	64.58 ± 0.42	[−0.15, 0.35]	0.428
S(1, −1)SumVarnc	283.70 ± 39.46	283.98 ± 45.21	283.31 ± 29.87	[−16.43, 17.78]	0.938
Skewness	−0.40 ± 0.94	−0.38 ± 0.97	−0.43 ± 0.91	[−0.35, 0.46]	0.788
Teta4	0.01 ± 0.10	0.01 ± 0.11	−0.00 ± 0.10	[−0.03, 0.06]	0.530
S(2, −2)SumEntrp	1.77 ± 0.05	1.77 ± 0.06	1.78 ± 0.03	[−0.03, 0.01]	0.331
S(5, 5)Correlat	0.10 ± 0.08	0.12 ± 0.09	0.08 ± 0.06	[−0.00, 0.07]	0.070
Teta2	−0.32 ± 0.07	−0.32 ± 0.07	−0.31 ± 0.06	[−0.04, 0.01]	0.312
HorzlRLNonUni	11100.81 ± 3419.84	11213.40 ± 3164.07	10938.18 ± 3799.62	[−1206.14, 1756.59]	0.713
Variance	251.37 ± 145.23	266.29 ± 166.00	229.83 ± 107.08	[−26.02, 98.92]	0.249
Teta3	0.53 ± 0.10	0.53 ± 0.11	0.53 ± 0.10	[−0.05, 0.04]	0.913
S(3, 3)SumOfSqs	98.65 ± 12.76	98.01 ± 14.08	99.58 ± 10.69	[−7.09, 3.95]	0.572
Sigma	0.59 ± 0.09	0.58 ± 0.10	0.60 ± 0.09	[−0.06, 0.02]	0.344
WavEnLH_s-3	197.88 ± 67.80	192.23 ± 65.37	206.05 ± 71.31	[−43.06, 15.43]	0.350

*Note*. Data were presented as mean ± standard deviation. ^*∗*^*p* < 0.05; ^*∗∗*^*p* < 0.01.

## Data Availability

The data used to support the findings of this study are available from the corresponding author upon request.
